# A Systematic Review of Apps using Mobile Criteria for Adolescent Pregnancy Prevention (mCAPP)

**DOI:** 10.2196/mhealth.6611

**Published:** 2016-11-10

**Authors:** Elizabeth Chen, Emily Rose Mangone

**Affiliations:** ^1^ Department of Health Behavior Gillings School of Global Public Health The University of North Carolina at Chapel Hill Chapel Hill, NC United States; ^2^ International Health Division Abt Associates Bethesda, MD United States; ^3^ Department of Health Policy and Management Gillings School of Global Public Health The University of North Carolina at Chapel Hill Chapel Hill, NC United States

**Keywords:** mHealth, eHealth, smartphone, mobile phone, app, teen, adolescent, young adult, systematic review, unintended pregnancy, family planning, pregnancy prevention, contraception

## Abstract

**Background:**

Adolescents in the United States and globally represent a high-risk population for unintended pregnancy, which leads to high social, economic, and health costs. Access to smartphone apps is rapidly increasing among youth, but little is known about the strategies that apps employ to prevent pregnancy among adolescents and young adults. Further, there are no guidelines on best practices for adolescent and young adult pregnancy prevention through mobile apps.

**Objective:**

This review developed a preliminary evaluation framework for the assessment of mobile apps for adolescent and young adult pregnancy prevention and used this framework to assess available apps in the Apple App Store and Google Play that targeted adolescents and young adults with family planning and pregnancy prevention support.

**Methods:**

We developed an assessment rubric called Mobile Criteria for Adolescent Pregnancy Prevention (mCAPP) for data extraction using evidence-based and promising best practices from the literature. mCAPP comprises 4 domains: (1) app characteristics, (2) user interface features, (3) adolescent pregnancy prevention best practices, and (4) general sexual and reproductive health (SRH) features. For inclusion in the review, apps that advertised pregnancy prevention services and explicitly mentioned youth, were in English, and were free were systematically identified in the Apple App Store and Google Play in 2015. Screening, data extraction, and 4 interrater reliability checks were conducted by 2 reviewers. Each app was assessed for 92 facets of the mCAPP checklist.

**Results:**

Our search returned 4043 app descriptions in the Apple App Store (462) and Google Play (3581). After screening for inclusion criteria, 22 unique apps were included in our analysis. Included apps targeted teens in primarily developed countries, and the most common user interface features were clinic and health service locators. While app strengths included provision of SRH education, description of modern contraceptives, and some use of evidence-based adolescent best practices, gaps remain in the implementation of the majority of adolescent best practices and user interface features. Of the 8 best practices for teen pregnancy prevention operationalized through mCAPP, the most commonly implemented best practice was the provision of information on how to use contraceptives to prevent pregnancy (15/22), followed by provision of accurate information on pregnancy risk of sexual behaviors (13/22); information on SRH communication, negotiation, or refusal skills (10/22); and the use of persuasive language around contraceptive use (9/22).

**Conclusions:**

The quality and scope of apps for adolescent pregnancy prevention varies, indicating that developers and researchers may need a supportive framework. mCAPP can help researchers and developers consider mobile-relevant evidence-based best practices for adolescent SRH as they develop teen pregnancy prevention apps. Given the novelty of the mobile approach, further research is needed on the impact of mCAPP criteria via mobile channels on adolescent health knowledge, behaviors, and outcomes.

## Introduction

The prevention of unintended pregnancy among adolescents and young adults is a health priority in the United States and globally because of its substantial health, social, and economic impacts [1-3]. Factors cited as key components to preventing teen pregnancy include free access to information, access to reproductive health services, and sex education [4-6]. The technology that has the potential to connect adolescents to these interventions and services is rapidly proliferating. In the United States, smartphone ownership among teens has grown exponentially, from 37% in 2013 to 73% in 2015, and almost all teens (91%) go online from a mobile device at least occasionally [7-9]. Access to smartphones in the United States is not limited to the wealthy; 61% of teens in households with annual incomes of less than $30,000 have access to smartphones [7]. Additionally, black and Hispanic teens, groups that experience 2 to 3 times the rate of unintended pregnancy as white teens, have the same or higher access to smartphones compared to white teens (85%, 71%, and 71%, respectively) [7,10]. While smartphones are currently more common in developed country settings, globally, smartphone subscriptions are projected to increase from 2.1 billion subscriptions in 2015 to 6.1 billion in 2020, covering 70% of the world’s population [11].

Mobile phone apps present a unique opportunity to connect teens to contraceptive information and sex education, behavior change interventions, and reproductive health services that could not be achieved previously. A recent review on apps for the prevention of unintended pregnancy in the general population found 218 apps that claimed to help prevent unintended pregnancy [12]. However, only 16 of these apps specifically targeted young adults, and the criteria used to evaluate these apps were developed for a broader population, without special consideration for adolescents [13].

Mobile health (mHealth), the use of mobile phones and other wireless technology in health care, is a burgeoning field within public health, and there is a need to standardize how mHealth interventions are developed, evaluated, and reported so that the field can advance. Recently, the mHealth Technical Evidence Review Group of the World Health Organization (WHO) developed the mHealth Evidence Reporting and Assessment (mERA) checklist to give guidance for reporting mHealth interventions, and a recent study implemented the mERA checklist to evaluate the reporting of mobile adolescent sexual and reproductive health interventions [14,15]. While adoption of mERA will lead to greater transparency and consistency in reporting across the broader field of mHealth, there is a need to develop nuanced and evidence-based checklists for specific mHealth approaches, such as adolescent pregnancy prevention and the promotion of sexual and reproductive health through mobile channels. Recognizing this gap, we build on the methodology and findings of a previous review of mobile apps for pregnancy prevention and identify evidence-based best practices for adolescent and young adult pregnancy prevention from the literature to develop and implement a preliminary framework for mobile Criteria for Adolescent Pregnancy Prevention (mCAPP) [12].

Using mCAPP, we conducted a systematic analysis of apps explicitly targeting adolescents and young adults and posed the following research questions:

1. What are the characteristics of teen pregnancy prevention     apps currently on the market?

2. What features are included in the user interface of these apps?

3. What teen pregnancy prevention best practices are used?

4. What sexual and reproductive health features and content are     offered?

Our review presents the answers to these questions and offers mCAPP as a foundation for future research in this field. Finally, we offer recommendations to app developers and researchers developing mobile phone apps for the prevention of unintended pregnancy among adolescents and young adults.

## Methods

### Codebook and Mobile Criteria for Adolescent Pregnancy Prevention Checklist Development

The codebook and data extraction form were developed iteratively between June and August 2015 and were informed by literature on mHealth, eHealth (electronic health information and communication technology, of which mHealth is a subset characterized by its mobile channels), and evidence-based adolescent reproductive health best practices (cited individually below). We established 4 domains: general app information, user interface, teen pregnancy prevention best practices for mobile apps, and general sexual and reproductive health features and content. Data extraction points were created in each domain, and data were collected and analyzed in Excel (Microsoft Corp). The codebook included 92 data extraction points for every app. To create a user-friendly mCAPP checklist (see Multimedia Appendix 1) for general use, we condensed some of the separate binary data extraction points (eg, inclusion of 7 race/ethnicity categories for images) into an easily digestible, 2-page format that remains organized around the 4 domains from our codebook.

### Search and Screening Strategy

In July 2015, we developed a set of 52 search terms informed by 3 previous systematic reviews on adolescent sexual health by combining 4 age-targeted terms, “adolescent,” “teen,” “young adult,” and “youth,” with 13 pregnancy prevention terms: “pregnancy prevention,” “sex education,” “sexual negotiation,” “family planning,” “sexual health,” “reproductive health,” “abstinence,” “sexual communication,” “sexual decision making,” “sexuality,” “condom,” “contraception,” and “birth control” [12,16,17]. Each of these 52 terms was searched separately by both authors in the Apple App Store and the Android Google Play store, and the number of results per search term was documented. Since Android and iOS (Apple) phones make up over 95% of the worldwide smartphone market share, the authors chose to review apps only in the relevant two app stores [18]. All app descriptions were screened for inclusion and exclusion criteria just as abstracts are initially screened in systematic reviews (Textbox 1).

**Inclusion and exclusion criteria.**

Inclusion criteria:

- App advertises and includes a component of pregnancy prevention, including sexual decision making information, contraception information that explicitly notes pregnancy prevention, or communication/negotiation information.
- App explicitly targets “adolescents,” “teens,” “young adults,” or “youth”or “students” in title or app store description.
- App is in English.
- App is free.

Exclusion criteria:

- App is primarily for the purpose of facilitating or tracking an established pregnancy.
- App is primarily a birth control reminder or fertility tracker with minimal additional information.
- App is primarily a locator (for clinics, condoms, etc).
- App is primarily information about hours, locations, and services provided by brick-and-mortar centers or clinics.
- App is for a specific event such as a conference, march, or rally.
- App primarily addresses infertility or in vitro fertilization.
- App primarily promotes pro-life viewpoints or opinions with no component of pregnancy prevention.
- App primarily targets adults.
- App is primarily a condom game that is not explicitly for educational purposes.
- App includes screenshots or descriptions that are not in English (even if English is listed as a language), is in beta testing, contains pranks, or is dysfunctional.
- Google Play app is a duplicate of a relevant app in the Apple App Store (Google Play app excluded, Apple App Store app included).

Apps that were primarily fertility trackers, birth control reminders, or locators were excluded because they were evaluated thoroughly in a recent systematic review and have too narrow of a purpose to benefit from an evaluation that takes a holistic approach [12]. Apps that were available in both the Apple App Store and the Google Play store were removed from the Google Play group and only analyzed as Apple App Store apps to avoid duplication. Full apps were downloaded for data abstraction.

### Relevance Review and Data Extraction

Two rounds of interrater reliability (IRR) calculations were conducted during the initial screening of app descriptions. Both authors documented the number of results they had for each search term and then compared the list of apps that met the inclusion criteria from each search term. IRR was 99% in both round 1 and round 2. IRR scores were high because of the low sensitivity of the Apple App Store and Google Play search engines and the resulting high volume of unrelated apps. Relevant apps were downloaded using an Android phone and an iPhone, and data extraction occurred in September 2015. Two additional rounds of IRR checks were done for the 92 data extraction points for 22% (8/37) of the downloaded apps, with IRR scores of 90% in round 3 and 96% in round 4.

### Characteristics of Apps

To classify apps and describe the general characteristics of apps available for prevention of unintended pregnancy among adolescents, we documented data from the app stores such as developer name, number of average stars, date last updated, app store category, and age rating; evaluated the race/ethnicity featured in the app images; determined which gender the app targeted; documented whether the app was affiliated with a specific religion; evaluated app credibility; evaluated app abortion stance; and evaluated the primary purpose of app (education, linkage to care, counseling support). We included characteristics that teens may use to determine credibility of source, peer use and approval, and fit with personal need or demographics [19]. There were 25 data extraction points in this section in the codebook.

### User Interface Features

To describe the design, interactivity, and engagement of included apps, we reviewed apps for 15 useful interface features. These features were informed by the literature on teen and young adult user interface preferences and marketing as well as criteria for evaluating health websites (Textbox 2) [19-21]. 

We also evaluated apps for the presence of 4 undesirable interface components (Textbox 3).

**Positive user interface features.**

□ Global positioning system (GPS) capabilities

□ Clinic or service locators

□ Contraceptive locators

□ Customizable look

□ Appointment scheduling

□ Public, forum, or social network communication

□ Direct communication (chat, text, call)

□ Push notifications

□ Internal search function

□ Videos, films, or movies

□ Gamification elements

  ○ Quizzes

  ○ Direct manipulation

□ Decision aid

□ Other notable features

**Negative user interface features.**

□ Faulty element (eg, broken links, blank pages, or broken/unintelligible English)

□ App crashed

□ Advertising

□ App required purchase to use

### Teen Pregnancy Prevention Best Practices for Apps

We were most interested in teen pregnancy prevention best practices relevant for mobile platforms, specifically apps. Because there are no current best practices for mHealth or eHealth interventions for teen pregnancy prevention, we consulted experts in the field and reviewed and operationalized sets of best practices in the peer-reviewed literature for in-person teen pregnancy prevention interventions that could reasonably be expected to succeed via this new mode of intervention delivery. Best practices that were specific to traditional classroom-based or other in-person teen pregnancy interventions and outside of the control of app developers were excluded. Upon review of the literature, we developed the following list of best practices as an evaluation framework for current apps (Textbox 4) [22-26]. As with general teen pregnancy interventions, it is ideal for teen pregnancy apps to use all 8 of these best practices.

**Best practices for app-based mHealth interventions for teen pregnancy prevention.**

□ Deliver and consistently reinforce persuasive communication about abstaining from sexual activity [22-24]

□ Deliver and consistently reinforce persuasive communication about using condoms or other forms of contraception when sexually active [22-24]

□ Be based on theoretical approaches that have been demonstrated to influence other health-related behavior and identify specific important sexual     antecedents to be targeted [22,23,25,26]

□ Provide clear, accurate information about the risk of pregnancy due to sexual activity [22,23]

□ Provide clear, accurate information and skill-building exercises on how to use contraceptives to prevent unwanted pregnancy [22,23]

□ Provide skill-building exercises or practice with sexual communication, negotiation, and refusal [22,24]

□ Provide activities designed to engage users, personalize or internalize information, and provide tailored feedback [22,24]

□ Target information for racial and ethnic subgroups of adolescents (eg, Hispanic females) [25,26]

**Promising practices for adolescent pregnancy prevention.**

□ Address whether parental consent is required for sexual and reproductive health services [27-30]

□ Encourage parental communication [31]

□ Offer peer sexual and reproductive health stories or peer counseling [32-34]

We also found 3 practices that, while not currently hailed as best practices in the literature, were cited by multiple sources as promising practices given the specific challenges that adolescents face in the context of reproductive health (Textbox 5).

### General Sexual and Reproductive Health Features and Content

WHO defines sexual and reproductive health (SRH) as a state of physical, emotional, mental, and social well-being in all matters relating to sexuality and the reproductive system and its processes. WHO adds that this state “implies that people are able to have a satisfying and safe sex life and that they have the capacity to reproduce and the freedom to decide if, when, and how often to do so” [35]. For our final research question about general SRH features and content, we organized the data into 2 subsections: one that included 7 SRH features relevant for all ages (Textbox 6) and one that focused on the provision of contraceptive information (Textbox 7). Both subsections were adapted from the SRH evaluation criteria developed in the earlier review [12].

In the contraceptive information subsection, we reviewed for the inclusion of 6 modern contraceptive methods, their effectiveness, and how to use or length of effectiveness of the specific method. We also reviewed for inclusion of related information. There were 37 data extraction points in this section in the codebook.

**Sexual and reproductive health features relevant for all ages.**

□ State that app is not a replacement for professional medical advice

□ Mention the confidentiality or privacy of the app or services accessed through the app

□ Address the cost of SRH services

□ Describe or counsel on abusive relationships or intimate partner violence

□ Describe or counsel on alcohol or substance abuse

□ Refer for pregnancy testing

□ Provide information about or refer for abortion services

**Sexual and reproductive health contraceptive information.**

□ Information on condoms, pills, injection, implant, intrauterine device (IUD), emergency contraception, withdrawal, or other method

□ Information on where the user can get contraceptives

□ Information/counseling about contraceptive side effects and risks

□ Information/counseling on side effect management

□ Information/counseling on switching contraceptive methods

□ Information/counseling on dual protection from pregnancy and sexually transmitted infections (STIs) including HIV

□ Information/counseling on STIs and STI testing

□ Notable misinformation or bad practices in the app (such as shaming or scare tactics)

## Results

### Search Results

**Figure 1:**
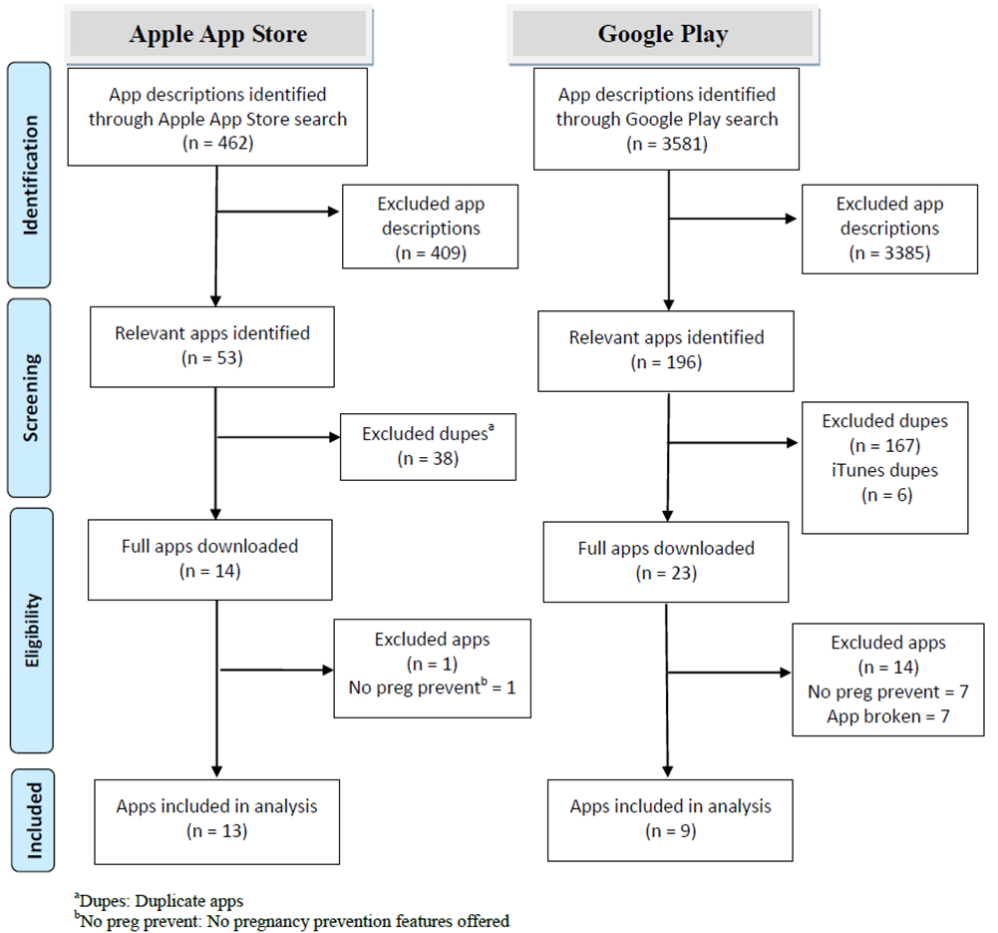
Preferred Reporting Items for Systematic Reviews and Meta-Analyses (PRISMA) diagram [36] for Apple App Store and Google Play search results.

Our search returned 4043 app descriptions from the Apple App Store (462) and Google Play (3581). Of these, 22 unique apps met inclusion criteria and were included in the review (Figure 1). Because of the poor sensitivity of the app store search engines and the hundreds of returned apps that were unrelated to reproductive health as well as the fact that it is not possible to export or copy information from the Apple App Store and Google Play, it was not feasible to document all reasons for exclusion at the app description level.

### Characteristics of Apps

Of the 22 included apps, 8 were available through the Apple App Store only, 9 were available through Google Play only, and 5 were available through both app stores. The 22 included apps were listed under a variety of app store categories: Health & Fitness (8/22), Education (5/22), Lifestyle (6/22), Reference (1/22), Medical (1/22), and Entertainment (1/22). The majority of the apps targeted adolescents in the United States (8/22) or the United Kingdom (7/22). The other apps focused on adolescents in Kenya (1/22), Africa (1/22), Pakistan (1/22), and Canada (1/22). Only 3 apps did not have a discernable geographic focus. A total of 13 apps included images of teens or young adults, and of these, 4 apps included images of white teens only, 2 apps included images of black teens only, and 7 apps included images of teens from more than one race. Almost all (21/22) apps provided information that was relevant for both males and females; 1 app specifically targeted females. Almost half (10/22) of the apps were downloaded less than 1000 times, 4 apps were downloaded 1000 to 10,000 times, and 8 apps did not have this information. See Multimedia Appendix 2 for general characteristics of included apps.

A total of 17 of the apps cited credible information from a reputable public health source (ie, Centers for Disease Control and Prevention or other health organization). The source of information in 5 apps was unclear or not credible. Many (15/22) of the apps had been updated in the past 12 months; all were updated within the past 24 months. We also evaluated each app’s abortion stance: 15 apps were categorized as pro-choice, 2 were categorized as pro-life, and 5 were categorized as unclear or no information. No apps were found to have an affiliation with a religion. Finally, we assessed each app’s apparent purpose(s). Categories were not mutually exclusive, and the overwhelming majority of the apps provided SRH education (19/22). Many also provided linkage to care (13/22) and counseling or support (12/22).

### User Interface Features

In evaluating the apps for desirable user features, we found that the most common features included in the apps were clinic and service locators (12/22) (see Table 1). Less than half (9/22) had global positioning system (GPS) capabilities; 7 of these 9 apps located contraceptives. Less than a quarter of the included apps offered entertainment, gamification, or communication features (5/22, 5/22, and 4/22, respectively). No apps allowed users to customize their design or schedule appointments.

With regard to the undesirable user interface features that we evaluated, we encountered faulty elements with 5 apps, advertisements with 3 apps, and crashing with 1 app. None of the apps required in-app purchases.

### Teen Pregnancy Prevention Best Practices for Apps

Of the 8 best practices for teen pregnancy prevention that we operationalized for mHealth interventions, the most commonly implemented best practice was the provision of information on how to use contraceptives to prevent pregnancy (15/22); followed by provision of accurate information on pregnancy risk of sexual behaviors (13/22); information on SRH communication, negotiation, or refusal skills (10/22); and the use of persuasive language around contraceptive use (9/22) (see Table 2). Among the 22 apps, *my choice by PPT* included 5 of the 8 best practices and one promising practice (parental consent). *Safe Sex Tips* also had 5 best practices but no promising practices. *Get S.M.A.R.T.* had 4 best practices and 2 promising practices. A total of 3 apps did not include any of the best or promising practices. No apps referenced a theoretical approach or encouraged parental communication, only 2 apps included targeting for racial/ethnic groups, and only 3 apps included persuasive language on abstinence.

### General Sexual and Reproductive Health Features and Contraceptive Content

Of the 7 general SRH features, 6 apps included 4 or more (Table 3). *my choice by PPT* again led with the highest number (6/7) of included SRH features. A total of 3 apps, *ASK My Body App, Healthwise*, and *ICAH*, did not include any of the SRH features. The most frequently offered SRH feature was the description of abortion options or counseling (12/22), followed by pregnancy testing referral, a statement of confidentiality, and information about abusive relationships (10/22 each). The least frequently provided SRH feature was a note that the app was not a replacement for medical advice (4/22) and information about alcohol or substance abuse (5/22).

Almost all apps (20/22) provided some information about male condoms as a way to prevent unintended pregnancy, and 17 apps mentioned condoms in the context of dual protection (from pregnancy and STIs). Only half (10/20) of apps that mentioned condoms also provided any information about how to use condoms. Emergency contraception was mentioned in 15 apps, followed by oral contraception (14/22), the intrauterine device (IUD) (14/22), and other methods (14/22). One-half to two-thirds of the apps that mentioned the condom, IUD, injection, and implant also described the effectiveness of the method at preventing pregnancy. Almost all of the apps that described long-acting methods, including the IUD, injection, and implant, also described the length of duration of the methods (13/14, 11/12, and 9/10, respectively). Almost all (14/15) apps that provided information about emergency contraception also provided some information about how to use it. Most (12/14) apps that provided information about oral contraception provided information about how to use it.

A total of 14 apps provided information on where to get contraceptives, and 10 apps provided information about side effects of using contraceptives. However, only 3 of these apps provided information about side effect management and switching methods. Most apps (19/22) provided information about STIs, and 15 provided information about getting tested for STIs.

**Table 1:** Desirable user interface features found in apps.

App name	Desirable user interface features												
	GPS^a^	CLI^b^	CON^c^	CUS^d^	SCH^e^	FOR^f^	DIR^g^	PUSH^h^	SEA^i^	VID^j^	GAM^k^	DA^l^	Total
*Get S.M.A.R.T.*	✓	✓				✓	✓	✓	✓	✓	✓	✓	9
*gPower*	✓	✓	✓				✓	✓		✓	✓	✓	8
*my choice by PPT*	✓	✓	✓						✓		✓	✓	6
*Teens in NYC*	✓	✓	✓					✓		✓			5
*Love Matters*		✓				✓			✓			✓	4
*NeedTayKnow*	✓	✓				✓						✓	4
*Your Choice Your Voice*	✓	✓	✓									✓	4
*CaSH 2 U*	✓	✓									✓		3
*Kent C Card*	✓	✓	✓										3
*The Choice—It's kind of a big deal*						✓			✓	✓			3
*Girls Incorporated of Lynn*		✓	✓										2
*OC Teens Mobile*		✓										✓	2
*SexPositive*										✓	✓		2
*SafeSex101*		✓	✓										2
*The Real Deal*	✓											✓	2
*aSk UK*							✓						1
*ICAH*								✓					1
*ASK My Body App*													0
*HealthWise*													0
*My Sex Doctor*													0
*My Sex Doctor Lite*													0
*Safe Sex Tips*													0
Total	9	12	7	0	0	4	3	4	4	5	5	8	

^a^GPS: Global positioning system

^b^CLI: Clinic or service locator

^c^CON: Contraceptive locator

^d^CUS: Customizable look

^e^SCH: Appointment scheduler

^f^FOR: Forum or social network

^g^DIR: Direct communication (text, call, or chat)

^h^PUSH: Push notifications

^i^SEA: Search function

^j^VID: Videos, films, or movies

^k^GAM: Gamification elements

^l^DA: Decision aid

**Table 2:** Adolescent pregnancy prevention best practices found in apps.

App name	Adolescent pregnancy prevention best practices										
	ABS^a^	CON^b^	THY^c^	PRI^d^	CI^e^	NEG^f^	PER^g^	TAR^h^	PAR^i^	COM^j^	PS^k^	Total
*Get S.M.A.R.T.*		✓		✓	✓	✓			✓		✓	6
*my choice by PPT*		✓		✓	✓	✓	✓		✓			6
*gPower*		✓		✓	✓		✓		✓			5
*Safe Sex Tips*		✓		✓	✓	✓	✓					5
*The Choice—It's kind of a big deal*	✓			✓		✓	✓				✓	5
*Your Choice Your Voice*	✓	✓			✓		✓		✓			5
*Love Matters*				✓	✓	✓		✓				4
*OC Teens Mobile*	✓			✓	✓	✓						4
*SexPositive*					✓	✓	✓				✓	4
*The Real Deal*		✓		✓	✓	✓						4
*ASK My Body App*					✓		✓	✓				3
*aSk UK*		✓		✓	✓							3
*My Sex Doctor*				✓	✓	✓						3
*My Sex Doctor Lite*				✓	✓	✓						3
*NeedTayKnow*		✓		✓	✓							3
*Teens in NYC*					✓				✓		✓	3
*Girls Incorporated of Lynn*		✓		✓								2
*CaSH 2 U*									✓			1
*ICAH*											✓	1
*HealthWise*												0
*Kent C Card*												0
*SafeSex101*												0
Total	3	9	0	13	15	10	7	2	6	0	5	

^a^ABS: Pro-abstinence messaging

^b^CON: Pro-contraception messaging

^c^THY: Noted a theoretical approach

^d^PRI: Pregnancy risk information

^e^CI: Contraceptive use information

^f^NEG: Communication, negotiation, and refusal skill information

^g^PER: Personalization

^h^TAR: Targeting of information for ethnic or racial groups

^i^PAR: Parental consent for sexual and reproductive health (SRH) services information

^j^COM: Parental communication encouragement

^k^PS: Peer stories or peer counseling

**Table 3:** Sexual and reproductive health features found in apps.

App name	Sexual and reproductive health features							
	DIS^a^	CON^b^	COS^c^	VIO^d^	SUB^e^	TES^f^	ABO^g^	Total
*my choice by PPT*	✓	✓	✓	✓		✓	✓	6
*aSk UK*		✓	✓	✓	✓		✓	5
*Get S.M.A.R.T.*		✓	✓	✓		✓	✓	5
*NeedTayKnow*		✓	✓	✓	✓		✓	5
*Your Choice Your Voice*		✓	✓	✓		✓	✓	5
*CaSH 2 U*		✓	✓			✓	✓	4
*Love Matters*				✓		✓	✓	3
*My Sex Doctor*				✓		✓	✓	3
*My Sex Doctor Lite*				✓		✓	✓	3
*OC Teens Mobile*	✓			✓	✓			3
*SafeSex101*			✓			✓	✓	3
*The Choice—It's kind of a big deal*		✓		✓	✓			3
*The Real Deal*	✓					✓	✓	3
*Girls Incorporated of Lynn*						✓	✓	2
*gPower*	✓		✓					2
*Kent C Card*		✓	✓					2
*Safe Sex Tips*					✓			1
*SexPositive*		✓						1
*Teens in NYC*		✓						1
*ASK My Body App*								0
*HealthWise*								0
*ICAH*								0
Total	4	10	9	10	5	10	12	

^a^DIS: Medical advice disclaimer

^b^CON: Statement of confidentiality

^c^COS: Service cost information

^d^VIO: Violence or abuse information

^e^SUB: Substance abuse information

^f^TES: Pregnancy testing referral available

^g^ABO: Abortion counseling or information

## Discussion

### Principal Findings

The 22 reviewed apps employed a variety of strategies to assist teens and adolescents in preventing unintended pregnancies. Overall strengths of included apps were the provision of SRH education, descriptions of at least 1 modern contraceptive method, and the use of 4 or more of the adolescent pregnancy prevention best or promising practices. It was also promising that 10 of the 22 apps provided information or counseling around SRH communication, negotiation, and refusal skills.

As a group, the apps also had weaknesses. Only half included persuasive messages, indicating that many apps were not explicitly advocating for behavior change. Only 2 of the apps (*Love Matters* and *ASK My Body App*) included targeted information for a specific ethnic or racial group of adolescents. Additionally, none of the apps explicitly cited theory, so there is no evidence of an established change strategy in the development of the apps. *Safe Sex Tips* is the only app that included 6 of the 8 best practices we evaluated, but this app is also one that was categorized as having unclear or no credibility. Moreover, there were 5 apps that did not include any of the best practices that we evaluated. Given that there were only 22 apps included in this study, it is concerning that 5 apps do not offer any of the best practices for teen pregnancy prevention and 3 apps do not offer any best or promising practices. These findings indicate that current best practices are not being implemented in available apps for teen pregnancy prevention, possibly because there are no guidelines for developers in this field.

In terms of user interface, one approach that was underused with this demographic was gamification. Only 5 of the 22 apps employed gamification elements. Encouragingly, these 5 apps included promising features for engagement. For example, *SexPositive* featured an interactive spinning wheel that matched body parts (eg, mouth, vagina) with different body parts or objects (eg, penis, teddy bear) and then provided information on the resulting risk of sexually transmitted infections, recommended safer sex practices, and communication and advice. While using games for teen pregnancy prevention is not entirely new, using a mobile phone instead of a computer or console is [36-39]. Given that 59% of girls and 84% of boys ages 13 to 17 years play video games online or on their phones, gamification may be a promising strategy to explore for future teen pregnancy prevention apps [40].

One factor that may limit the impact of the 22 teen pregnancy prevention apps was that 19 of them were location- or organization-specific. For example, *SafeSex 101* was developed for students at the University of California, Los Angeles. The app listed information on local resources and nearby health care providers and guided students on how to access birth control given the university’s specific insurance plans. While these apps may provide a lot of relevant information, adolescents searching for these apps may assume that these apps do not apply to them simply because they are not part of the app’s target demographic. Because of GPS, apps can provide global and local services based on the user’s location. More apps are needed that follow best practices and include resources for national and even international audiences.

### Limitations

There are limitations to this review. Because there is not a list of best practices for mHealth-based teen pregnancy prevention interventions, we synthesized and adopted existing best practices from the literature with a critical eye for the strengths and limitations of mobile phones, creating mCAPP. This ambitious 92-item checklist represents a novel and important foundation for developing and evaluating adolescent sexual and reproductive health interventions on a mobile platform. However, the validity and effectiveness of mCAPP as a cohesive set of criteria has not yet been evaluated for this platform, and mCAPP will require further study to understand its impact in practice. As it stands, this checklist is intended to help developers and evaluators alike consider best practices for adolescent SRH in the context of mobile apps where previously there was little guidance. Future studies are needed to determine the impact of the mCAPP criteria and whether there are synergistic effects between different criteria that lead to improved outcomes for adolescents.

Another limitation is that while we sought to assess whether apps were based on theory, we only evaluated whether a theory was explicitly cited. Therefore, some apps may have had theoretical underpinnings that went undocumented. One technical limitation was that we tried to only evaluate content available in the app (assuming that data plans and Internet connection may not always be available) and not consider secondary content (YouTube videos, external links). However, it was occasionally difficult to make this distinction when content was embedded in the app. When in doubt, we reviewed inclusively. A final limitation is that by the time this article is published, there will almost certainly be more apps available at the Apple App Store and Google Play that would fit our inclusion criteria and propose innovative strategies to address adolescent pregnancy on this platform.

### Conclusion

In this quickly evolving field and market, we must assess the strategies that are used to prevent unintended pregnancy among youth through mobile phones and lay groundwork for more effective interventions that emphasize behavior change as well as knowledge dissemination. We put forth mCAPP as a preliminary framework that can guide development and evaluation efforts for developers and researchers alike. Additional research should be conducted to assess the impact of apps that leverage mCAPP strategies compared to apps that do not on reproductive and sexual health knowledge, behaviors, and outcomes of adolescents. With more and more adolescents accessing smartphones, our hope is that mCAPP can serve as a framework and catalyst for new mHealth interventions that can revolutionize mobile health, starting with adolescent pregnancy prevention.

## Appendix

Multimedia Appendix 1 Mobile Criteria for Adolescent Pregnancy Prevention checklist.

Multimedia Appendix 2 Overview of apps included in review.

## Abbreviations

eHealth: electronic health information and communication technology
GPS: global positioning system
IRR: interrater reliability
IUD: interuterine device
mCAPP: Mobile Criteria for Adolescent Pregnancy Prevention
mERA: mHealth Evidence Reporting and Assessment
mHealth: mobile health
SRH: sexual and reproductive health
STI: sexually transmitted infection
WHO: World Health Organization
