# Supplementary material for: A Systematic Review of Apps using Mobile Criteria for Adolescent Pregnancy Prevention (mCAPP)
Source: JMIR Mhealth Uhealth. 2016 Nov 10;4(4):e122. doi: 10.2196/mhealth.6611 (PMC5122721; doi:10.2196/mhealth.6611)
Supplement: Multimedia Appendix 1 [file mhealth_v4i4e122_app1.pdf]

mCAPP - Mobile Criteria for Adolescent Pregnancy Prevention

FOR EVALUATORS

General App Information

Name of app ______________________________

Name of app developer______________________________

App store ______________________________

Current version______________________________

Current version average stars/rating______________________________

Current version number of reviews ______________________________

App store category ______________________________

Date last updated ______________________________

Number of installs (only available in Google Play) ______________________________

Age rating ______________________________

Sponsoring organization (if different from app developer) ______________________________

Target gender	☐ Male	☐ Female	☐ Both
Perceived race/ethnicity in images (choose all that apply)	☐ None (no images)<br>☐ Hispanic/Latino<br>☐ Unclear	☐ White<br>☐ Asian	☐ Black<br>☐ Native American
Religious affiliation	☐ Yes<br>If yes, name religion: ______________________________	☐ No	
Geographic affiliation	☐ Domestic (U.S.)<br>List cities/countries, if applicable: ______________________________	☐ International	☐ None
Source credibility	☐ Credible	☐ Not credible/unclear	
Abortion stance/tone	☐ Pro-choice	☐ Pro-life	☐ Unclear/neutral
App purpose (choose all that apply)	☐ Education	☐ Linkage to care	☐ Counseling/support

Summary of app purpose: ______________________________

FOR DEVELOPERS & EVALUATORS

User Interface – Desirable Features

- ☐ GPS
- ☐ Info on clinics/services nearby
- ☐ Info on where to get contraceptives nearby
- ☐ Appointment scheduling
- ☐ Customizable look or feel
- ☐ Public communication (e.g. forums)
- ☐ Direct communication (chat/messaging)
- ☐ Push notifications
- ☐ Search function
- ☐ Gamification elements
- ☐ Direct manipulation
- ☐ Quizzes
- ☐ Videos/films
- ☐ Decision aids

User Interface – Undesirable Features

- ☐ Faulty element
- ☐ App crashes
- ☐ Purchase requirement
- ☐ Advertisement(s)

Notable interface features: ______________________________

FOR DEVELOPERS & EVALUATORS

Teen Pregnancy Prevention Best Practices

- ☐ Persuasive language about abstinence (e.g. "teens *should…*", "most students do…")
- ☐ Persuasive language about contraception (e.g. "teens *should…*", "most students do…")
- ☐ Theoretical approach (i.e. theory is explicitly cited in app or app description)
- ☐ Accurate information about pregnancy risk of sexual activities
- ☐ Accurate information on how to use contraceptives
- ☐ Examples of or practice with sexual communication, negotiation, or refusal skills
- ☐ Activities designed to involve users and have them personalize the information
- ☐ Targeting for specific race/ethnic group

Teen Pregnancy Prevention Promising Practices

- ☐ Information on parental consent (e.g. "Parental consent is not required to obtain birth control in California")
- ☐ Encouragement of parental communication
- ☐ Peer connection (peer stories or peer counseling)

Sexual and Reproductive Features and Content

- ☐ Information on the cost of sexual and reproductive health (SRH) services
- ☐ Information about confidentiality and/or privacy of app and SRH services
- ☐ Professional medical advice disclaimer (e.g. "App is not a replacement for professional medical advice)
- ☐ Information or counseling on abusive relationships or violence
- ☐ Information or counseling on alcohol or substance abuse
- ☐ Information or counseling on pregnancy testing/referral
- ☐ Information or counseling on abortion options & services

Contraceptive Information:

☐ Male condoms<br>☐ How to use ☐ Effectiveness	☐ Oral pills<br>☐ How to use ☐ Effectiveness
☐ Injection (Depo)<br>☐ How to get/duration ☐ Effectiveness	☐ Implant<br>☐ How to get/duration ☐ Effectiveness
☐ IUD<br>☐ How to get/duration ☐ Effectiveness	☐ Emergency contraception<br>☐ How to use ☐ EC not abortion
☐ Withdrawal<br>☐ When to withdraw ☐ Effectiveness	

- ☐ Information on other contraceptive methods
- ☐ Information on where to get contraceptives
- ☐ Information on side effects of contraceptives
- ☐ Information or counseling on side effect management
- ☐ Information or counseling on switching method of contraceptives
- ☐ Information on dual protection of condoms (against pregnancy and STIs)
- ☐ Information on sexually transmitted infections (STIs)
- ☐ Information on STI testing

Notable information/bad practices: ____
