# Supplementary material for: A Systematic Review of Apps using Mobile Criteria for Adolescent Pregnancy Prevention (mCAPP)
Source: JMIR Mhealth Uhealth. 2016 Nov 10;4(4):e122. doi: 10.2196/mhealth.6611 (PMC5122721; doi:10.2196/mhealth.6611)
Supplement: Multimedia Appendix 2 [file mhealth_v4i4e122_app2.pdf]

SUPPLEMENTAL MATERIAL A. Overview of Apps Included in Review

App Store Availability	App Name	Developer/Sponsor	Category	Geographic Location Specified by App	Last Updated	Installs
iTunes	my choice by PPT	Planned Parenthood Toronto	Education	Toronto, Canada	1/20/2015	N/A
iTunes	SexPositive	University of Oregon	Education	University of Colorado Boulder, University of Maryland, University of Oregon	9/4/2014	N/A
iTunes	NeedTayKnow	John-Paul Thain/ National Health Service Tayside	Education	Tayside, UK	5/14/2015	N/A
iTunes	Safe Sex Tips	Dharmendra Solanki	Entertainment		1/26/2015	N/A
iTunes	Your Choice Your Voice	Thomas Benjamin Ltd/ Bromley Healthcare, National Health Service	Health & Fitness	London, UK	10/9/2014	N/A
iTunes	SafeSex101	Associated Students UCLA	Lifestyle	Los Angeles, California, United States	2/23/2013	N/A
iTunes	The Choice - it's kind of a big deal	Project Your Choice LLC	Lifestyle		8/22/2013	N/A
iTunes	aSk UK	John-Paul Thain/ National Health Service Tayside	Medical	Tayside, UK	2/24/2015	N/A
iTunes	Get S.M.A.R.T.	William Chavez/ Eight high school students	Reference	California	3/19/2015	N/A
GP	The Real Deal	Youth and Family Education Resources (YFER)/ Core of Life	Education		10/13/2013	100-500
GP	ICAH	ICAH	Education	Illinois, USA	11/6/2014	10-50

GP	ASK My Body App	Grappetite/ Youth Empowerment Alliance	Health & Fitness	Pakistan	8/10/2015	50-100
GP	Girls Incorporated of Lynn	The Middle School Pregnancy Prevention Program, and Teen Health Ambassador Program at Girls Incorporated of Lynn with funding from the MDPH Office of Adolescent Health and Youth Development	Health & Fitness	Lynn, Massachusetts, USA	10/21/2013	100-500
GP	OC Teens Mobile	AmongTheNationsMD/ CATCH, American Academy of Pediatrics	Health & Fitness	Orange County, CA	7/20/2013	100-500
GP	CaSH 2 U	ICE/National Health Service Wirral	Health & Fitness	UK	7/10/2013	100-500
GP	HealthWise	Noble Microsystems	Health & Fitness	Africa	10/2/2014	100-500
GP	Kent C Card	Kent Community Health NHS Trust	Health & Fitness	Kent, UK	9/11/2013	500-1000
GP	Love Matters	Kwetu Design/ Love Matters	Lifestyle	Kenya	3/19/2015	100-500
Both	gPower	Georgia Campaign for Adolescent Power & Potential	Health & Fitness	Georgia, USA	5/12/2015	1000-5000
Both	My Sex Doctor	MYSD LTD (National Health Service approved)	Lifestyle	UK	7/23/2015	5000-10000
Both	My Sex Doctor Lite	MYSD LTD (National Health Service approved)	Lifestyle	UK	7/23/2015	5000-10000
Both	Teens in NYC	NY Department of Health and Mental Hygiene	Lifestyle	New York City, USA	12/15/2014	5000-10000
